# Static CT myocardial perfusion imaging: image quality, artifacts including distribution and diagnostic performance compared to ^82^Rb PET

**DOI:** 10.1186/s41824-021-00118-x

**Published:** 2022-01-04

**Authors:** João R. Inácio, Sriraag Balaji Srinivasan, Terrence D. Ruddy, Robert A. deKemp, Frank Rybicki, Rob S. Beanlands, Benjamin J. W. Chow, Girish Dwivedi

**Affiliations:** 1 Department of Radiology, The Ottawa Hospital, University of Ottawa Research Institute, University of Ottawa, Ottawa, Canada; 2 Centro Hospitalar Universitário Lisboa Norte, Lisbon, Portugal; 3 Medical School, Harry Perkins Institute of Medical Research, The University of Western Australia, Perth, Australia; 4 Division of Cardiology, Department of Medicine, University of Ottawa Heart Institute, Ottawa, Canada; 5 Department of Radiology, University of Cincinnati, Cincinnati, OH USA; 6 Imagia, Montreal, QC Canada; 7 Department of Advanced Clinical and Translational Cardiovascular Imaging, Harry Perkins Institute of Medical Research and Fiona Stanley Hospital, Murdoch, Australia

**Keywords:** Computed tomography, Ischemia, Myocardial perfusion imaging, Rubidium positron emission tomography

## Abstract

**Background:**

Rubidium-82 positron emission tomography (^82^Rb PET) MPI is considered a noninvasive reference standard for the assessment of myocardial perfusion in coronary artery disease (CAD) patients. Our main goal was to compare the diagnostic performance of static rest/ vasodilator stress CT myocardial perfusion imaging (CT-MPI) to stress/ rest ^82^Rb PET-MPI for the identification of myocardial ischemia.

**Methods:**

Forty-four patients with suspected or diagnosed CAD underwent both static CT-MPI and ^82^Rb PET-MPI at rest and during pharmacological stress. The extent and severity of perfusion defects on PET-MPI were assessed to obtain summed stress score, summed rest score, and summed difference score. The extent and severity of perfusion defects on CT-MPI was visually assessed using the same grading scale. CT-MPI was compared with PET-MPI as the gold standard on a per-territory and a per-patient basis.

**Results:**

On a per-patient basis, there was moderate agreement between CT-MPI and PET-MPI with a weighted 0.49 for detection of stress induced perfusion abnormalities. Using PET-MPI as a reference, static CT-MPI had 89% sensitivity (SS), 58% specificity (SP), 71% accuracy (AC), 88% negative predictive value (NPV), and 59% positive predictive value (PPV) to diagnose stress-rest perfusion deficits on a per-patient basis. On a per-territory analysis, CT-MPI had 73% SS, 65% SP, 67% AC, 90.8% NPV, and 34% PPV to diagnose perfusion deficits.

**Conclusions:**

CT-MPI has high sensitivity and good overall accuracy for the diagnosis of functionally significant CAD using ^82^Rb PET-MPI as the reference standard. CT-MPI may play an important role in assessing the functional significance of CAD especially in combination with CCTA.

## Introduction

Cardiac computed tomography (CT) angiography (CCTA) is a noninvasive diagnostic method with high sensitivity and very high negative predictive value for the detection of obstructive coronary artery disease (CAD) in patients with chest pain (Budoff et al. 2008; Miller et al. 2008; Meijboom et al. 2008; Litt et al. 2012). However, both morphological data and functional significance of coronary stenosis are important for management and to impact clinical outcomes for symptomatic CAD patients considered for revascularization (Bruyne et al. 2012). Although CCTA can provide anatomical information regarding the presence of epicardial coronary artery stenosis, the hemodynamic significance of coronary stenosis usually requires assessment by catheter-based fractional flow reserve (FFR) (Meijboom et al. 2008; Pijls et al. 1996), or noninvasive methods such as single photon emission tomography/myocardial perfusion imaging (SPECT-MPI) (Gaemperli et al. 2008), positron emission tomography (PET) (Carli et al. 2007), cardiac magnetic resonance imaging (CMR) (Bettencourt et al. 2013), stress echocardiography (Jiménez-Navarro et al. 2001) and FFR_CT_ (FFR determined using CCTA images) (Pontone et al. 2019a).

CT-MPI has been compared with myocardial flow measured with microspheres (Eck et al. 2016), SPECT (Jaarsma et al. 2012; Cury et al. 2010; Tamarappoo et al. 2010), CMR (Jaarsma et al. 2012), FFR_CT_ (Pontone et al. 2019a; Yang et al. 2017) and invasive FFR (Jaarsma et al. 2012; Takx et al. 2015; Williams et al. 2017). Multiple experimental, single-centre and multi-centre clinical studies evaluated the diagnostic accuracy of CT myocardial perfusion (CT-MPI) to detect flow limiting stenosis and associated myocardial perfusion abnormalities in comparison with other methods (Pontone et al. 2019a; Eck et al. 2016; Jaarsma et al. 2012; Cury et al. 2010; Tamarappoo et al. 2010; Yang et al. 2017; Takx et al. 2015; Williams et al. 2017). CT-MPI is reported to have comparable diagnostic accuracy for detection of myocardial ischemia compared to invasive FFR (Takx et al. 2015). CT-MPI can be performed either using a static (obtains a single CT dataset at an estimated peak myocardial enhancement time after contrast administration) or a dynamic (multiple CT datasets obtained after contrast administration along a time-attenuation curve) method (Koo et al. 2016).

Rubidium-82 PET (^82^Rb PET) has been considered a noninvasive reference standard for the detection of obstructive CAD (Jaarsma et al. 2012; Mc Ardle et al. 2012), but there are limited studies that use Rb PET as a reference standard to assess CT-MPI, and no studies have used a static ECG-gated prospective acquisition protocol. The purpose of our study was to compare the diagnostic accuracy of rest/vasodilator stress static CT-MPI with vasodilator stress/rest ^82^Rb PET-MPI in identification of myocardial ischemia in patients with suspected CAD, using visual qualitative and semi-quantitative analysis. We also aimed to assess image quality and characterize artifacts in the CT-MPI studies.

## Methods

### Patients and study protocol

The study was approved by our institutional review board, and all patients provided written informed consent for inclusion and data analysis. Forty-four consecutive patients with clinically suspected or stable symptomatic CAD with a clinical indication for vasodilator stress/rest ^82^Rb PET-MPI at a single urban teaching hospital were prospectively enrolled to perform both rest/vasodilator stress CT-MPI and vasodilator stress/rest ^82^RbPET-MPI within 90 days. Patients with heart failure, acute coronary syndrome, reactive airway disease, chronic kidney disease, and allergy to iodinated contrast were excluded. No patient experienced any interval change in clinical status or coronary revascularization.

### CT-MPI protocol

#### Patient preparation

**Fig. 1:**
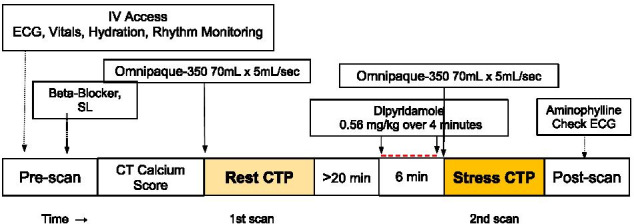
CT-MPI Protocol. After patient preparation and monitoring, non-contrast prospective ECG-triggered axial cardiac CT for calcium scoring was obtained. For CT-MPI, two consecutive rest/stress prospective ECG-triggered axial acquisitions were performed with a triphasic intravenous contrast medium injection protocol. After rest CT-MPI acquisition, a delay of at least 20 min to mitigate the effects of pre-medication with beta-blockers and nitroglycerine. Stress CT-MPI was acquired 2 min after completion of dipyridamole intravenous infusion. Once CT image acquisition was completed, intravenous aminophylline was administered to reverse the effects of dipyridamole

Participants abstained from caffeine for 12 h and from methylxanthine-containing products, oral dipyridamole, theophylline, and beta-blockers for 24 h before stress testing. An 18-gauge cannula was inserted into the right antecubital vein for stress agent and contrast administration. Before the rest CT acquisition, patients received metoprolol to target heart rate of < 65 beats/min and nitroglycerin (0.8 mg) sublingually. All patients were observed for one hour after the study (Fig. 1).

#### Image acquisition

A non-contrast prospective electrocardiogram (ECG) triggered axial (sequential) cardiac CT was performed for calcium scoring as per local clinical routine on a GE Volume CT (GE Healthcare, Waukesha, USA), with 64 × 0.625 mm collimation, gantry rotation 350 ms, with X-ray tube modulation, mA = 400–800, 120 kVp, at 70% RR interval (Chow et al. 2010). For CT-MPI, two consecutive prospective ECG-triggered axial acquisitions at 75% of R-R interval with ± 5% padding were performed with mA = 400–800, 100 kVp for body mass index < 30, 120 kVp for body mass index ≥ 30 (Abbara et al. 2016). A triphasic intravenous injection protocol (100% contrast, 40%/60% contrast/saline and 100% saline) of 70 mL of contrast medium (Omnipaque-350, GE Healthcare, Princeton, NJ, USA) was injected at 5 ml/s. For rest CT-MPI, acquisition was determined using a test bolus (20 ml of contrast) to determine time to peak in the ascending aorta. After a delay of at least 20 min, stress CT-MPI was acquired 2 min following dipyridamole injection which was infused intravenously at a dose of 0.14 mg/kg/min over 5 min (Cury et al. 2010). Stress CT-MPI was initiated using a real-time bolus tracking technique, at the time of peak left ventricle filling with contrast, which was determined with a threshold of 120 Hounsfield unit (HU). During dipyridamole infusion, heart rate, blood pressure, ECG, and symptoms were monitored continuously. Once image acquisition was completed, intravenous aminophylline (1.5 mg/kg body weight) was administered over 5 min to reverse the effects of dipyridamole.

#### CT-MPI image reconstruction

Prospectively acquired cardiac CT rest/stress data sets were reconstructed at 70, 75, and 80% of the R-R interval with a slice thickness of 0.625 mm, 250 mm reconstructed field of view, 512 × 512 matrix and standard reconstruction algorithm. Rest cardiac CT angiography images were used for clinical anatomic assessment of coronary stenosis. Rest/stress data sets for each patient were loaded into a dedicated workstation Aquarius iNtuition software (Version 4.4.11, TeraRecon, San Mateo, CA, USA) in multidata workflow allowing anatomical coregistration and synchronization of both data sets side by side for visual interpretation of myocardial attenuation in standard multiplanar images in short-axis, vertical-long-axis and horizontal long-axis of the left ventricle (LV), with 7 mm slice thickness images.

#### Interpretation of myocardial perfusion on CT-MPI

**Fig. 2:**
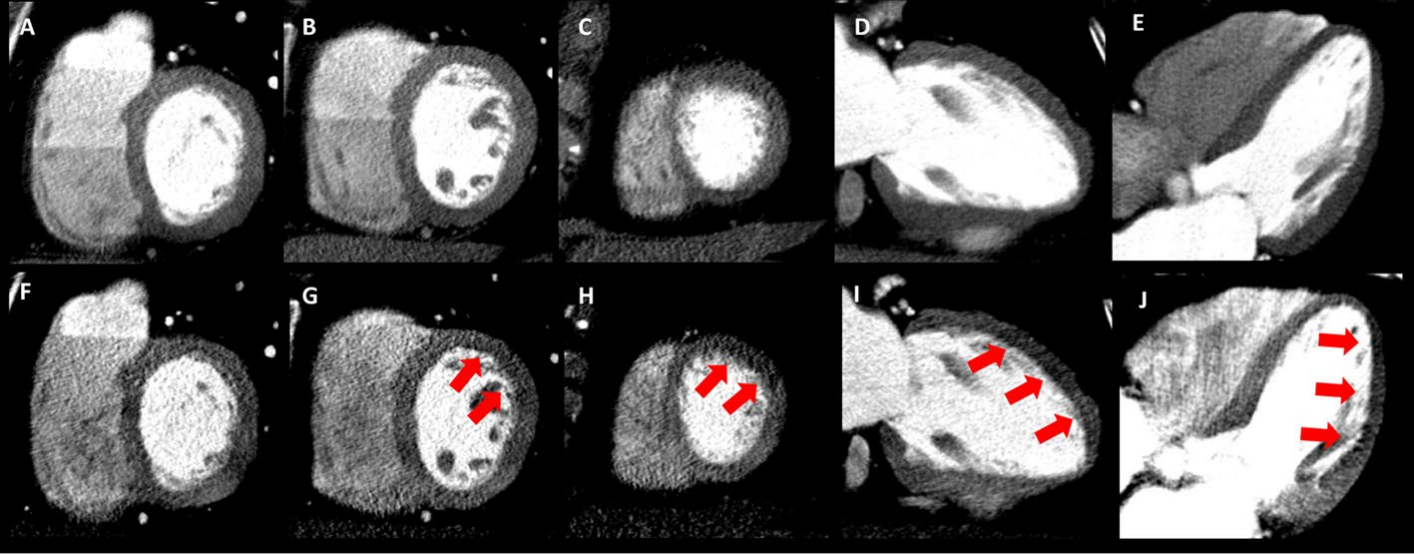
CT-MPI Rest-Stress visual analysis. CT-MPI in Rest (**A**–**E**) and Stress (**F**–**J**) in short axis, 2-chamber and 4-chamber multi-planar planes images with 5–7 mm slice thickness, display 200–300 HU window width and 100–150 HU window length. Perfusion defects on post dipyridamole stress CT-MPI in mid and apical anterior/anterolateral segments in a single patient (red arrows on **G**–**J**). The extent and severity of stress perfusion defect was scored as 1 for mid anterior segment, 2 mid anterolateral segment, 3 for apical anterior segment and 1 apical lateral segment. This corresponded to summed stress score of 7, summed rest score of 0 and sTPD 10%, iTPD 10%. *TPD* total perfusion deficit

Two experienced independent readers blinded to all clinical, CT coronary angiography and ^82^Rb PET findings analyzed and scored the CT-MPI studies. Individual data per reader and a combined consensus reading data were collected. Initial display window width and window level settings were 200 to 300 HU and 100 to 150 HU, respectively (Blankstein et al. 2009) but free manipulation of window width and window level settings was allowed. Regions of myocardium that exhibited significantly reduced CT attenuation as a result of decreased contrast distribution were considered to have abnormal perfusion. The attenuation of normal myocardium in each patient was used as an internal reference to visually compare and assess the relative severity of myocardial hypoperfusion (Fig. 2).

True perfusion defects were differentiated from potential artifacts by examining the morphology of myocardial perfusion defects and their relation with the LV endocardial contour. Perfusion defects that did not follow the endocardial contour or extended beyond the heart borders were considered artifacts. True perfusion defects were defined if present simultaneously in more than one plane (short-axis, vertical-long-axis and horizontal long-axis) and in two consecutive segments. Artifacts were registered according to the segments involved and classified as beam hardening, motion, step-artifact and noise. Image quality and reader’s confidence were registered using Likert scale 1 to 5 (1-inadequate to 5-excellent).

#### Visual assessment of perfusion deficit on CT-MPI

The extent and severity of perfusion defect in each segment was graded on a 0 to 4 scale (0 = uniform CT attenuation equal to normal myocardium; 1 = mildly reduced attenuation encompassing less than 25% of the myocardium; 2 = moderately reduced attenuation comprising 25% to 50% of the myocardium; 3 = moderately reduced attenuation in 50% to 75% of the myocardium; and 4 = severely reduced attenuation in more than 75% of the myocardium) (Fig. 2). The scores from both readers for each study were averaged to achieve a final perfusion deficit score for each study. The CT-MPI based summed stress score (SSS) and summed rest score (SRS) were derived by adding the scores assigned to each individual segment from stress and rest. A summed difference score (SDS) was defined as the difference between SSS and SRS. A visually assessed total perfusion deficit (TPD) for CT-MPI was derived by dividing CT-MPI SSS, SRS and SDS by 68 (which represents the sum of the maximum scores that could be assigned to each of the 17 segments) multiplied by 100%. The difference in TPD_stress_-TPD_rest_ was calculated representing the percentage of total myocardium involved with stress ischemia (iTPD), an approach previously applied to SPECT-MPI (Hachamovitch et al. 2003) and CT- MPI versus SPECT-CT comparison (Tamarappoo et al. 2010).

### ^82^Rb PET imaging

The ^82^Rb PET studies were performed according to our standard clinical protocol previously described (Ziadi et al. 2011). Patients refrained from caffeine ≥ 12 h and theophyllines for > 48 h before the ^82^Rb PET study. After an overnight fast, patients were positioned in a 3-dimensional PET system (Discovery Rx/VCT, GE Healthcare, Milwaukee, Wisconsin). A low-dose (0.25 mSv), fast helical (1.5 s) CT (120 kVp with axial and angular mA modulation at a noise index of 50) was acquired for attenuation correction. Then, 10 MBq/kg of ^82^Rb was administered intravenously over 30 s interval to ensure dead-time losses remained < 50% (RUBY-FILL®- rubidium chloride rb-82 injection solution, package insert, Jubilant DraxImage Inc). A 17-frame, 10-min dynamic ^82^Rb scan was acquired with a parallel list-mode acquisition.

After rest PET-MPI, a dipyridamole stress test was performed (0.14 mg/kg/min over 5 min). Then 10 MBq/kg of ^82^Rb was infused 3 min after completion of the vasodilator infusion. Stress images were acquired per rest MPI. A repeat low-dose CT scan was acquired after stress PET images for attenuation correction.

Images were reconstructed using Fourier rebinning and filtered back-projection with a 12-mm 3-dimensional Hann window of the ramp filter. The list-mode data from 2.5 to 10 min were replayed to reconstruct electrocardiographic-gated images. Images were interpreted using a 17-segment model and a 5-point scoring system (normal, mildly reduced, moderately reduced, severely reduced and absent ^82^Rb uptake) by an experienced reader blinded to clinical data. SSS, SRS and SDS were calculated semi-automatically (Cerqueira et al. 2002).

### Statistical analysis

Continuous variables were expressed as mean ± SD. Normally distributed continuous variables were compared using a paired t test and categorical variables with χ^2^ test. The presence of perfusion defect by ^82^Rb PET-MPI or CT-MPI was considered a categorical variable. Per-segment based, per-territory and per-patient comparison of abnormal segments by CT-MPI and ^82^Rb PET-MPI was expressed as sensitivity (SS), specificity (SP), accuracy (AC), positive predictive value (PPV), negative predictive value (NPV), positive likelihood ratio and negative likelihood ratio using ^82^Rb-MPI as the reference. Kappa estimates were used to evaluate the agreement between the 2 independent CT-MPI readers and between CT-MPI and ^82^Rb-MPI in identification of hypoperfused myocardial segments. Agreement between TPD by PET-MPI and visually derived TPD by CT-MPI were assessed using the Bland–Altman analysis by calculating the mean difference and SD of the differences. The range of ± 1.96 SD of the differences provided the 95% level of agreement between the two techniques. To further strengthen the comparison between the two techniques, the Passing-Bablok regression method was used to assess the significance of agreement. The significance of the linear agreement was analyzed with the CUSUM (cumulative summation) test for linearity; *P* < 0.05 indicated a significant consistent difference between the two techniques. Lastly, receiver operating characteristic (ROC) analysis (with interactive plot analysis) was used to test the predictive accuracy of CT-MPI with^82^Rb PET-MPI as the reference. Optimal cut-off was defined as the threshold where the sum of sensitivity and specificity was maximum.

## Results

### Patient and studies characteristics

Of the 44 patients (23 male) included in the analysis, 23 (52%) had history of CAD, and 23 (52%) were symptomatic with chest pain (Table 1). The interval period between CT-MPI and ^82^Rb PET studies was 20 ± 19 days (Table 2). Perfusion defects were detected by ^82^Rb PET in 18 patients, corresponding to a disease prevalence of 41%.

**Table 1:** Patients characteristics

Characteristic	Value
*n*	44
BMI	30.8 (± 6.7)
Age	61.7 (± 10.9)
Male/female	23 (52)/21 (48)
Past medical history of CAD:	
Previous MI	10 (23)
Previous PCI	10 (23)
Previous CABG	3 (7)
Smoking history	33 (75)
Diabetes	17 (39)
IDDM	4 (9)
NIDDM	13 (30)
Hypertension	35 (80)
Hyperlipidemia	36 (^82^)
Family History of CAD	24 (55)
Asymptomatic	9 (20)
New Chest Pain	
Typical	10 (23)
Atypical	12 (27)
Dyspnea	26 (59)
Palpitations	16 (36)
CCS class	
0	18 (41)
1	13(30)
2	4(9)
3	2(5)
4	7(16)
NYHA Class	
0	15(34)
1	13(30)
2	8(18)
3	1(2)
4	7(16)

Quantitative variables expressed as mean and ± standard deviation; categorical variables expressed as frequencies (percentages)

*BM*, body mass index; *CAD* coronary artery disease; *MI* myocardial infarct; *PCI* percutaneous coronary intervention; *CABG* coronary artery bypass graft surgery; *IDDM* insulin dependent diabetes mellitus; *NIDDM* noninsulin dependent diabetes mellitus; *CCS* Canadian Cardiovascular Society grading scale of angina; *NYHA* New York Heart Association functional classification

**Table 2:** Studies characteristics

Characteristic	Value
Duration in days between CT-MPI and ^82^Rb-PET MPI	20.3 (± 19.2)
Heart rate during rest CT-MPI (beats per minute)	54.2 (± 6.7)
Range of heart rate during rest CT-MPI (beats per minute)	39–67
Maximum heart rate during stress CT-MPI (beats per minute)	72.9 (± 8.9)
Range of heart rate during stress CT-MPI (beats per minute)	58–100
DLP of rest-stress CT-MPI, mGy/cm	598.1 (± 149.8)
Total effective radiation dose rest-stress CT-MPI, mSv	8.4 (± 2.1)
Total estimated effective radiation dose ^82^Rb-PET MPI, mSv	2

Average ± standard deviation; Effective dose = Dose length product (DLP) × 0.014mSV/mGy·cm

*CT-MPI* computed tomography myocardial perfusion imaging; ^*82*^*Rb PET MPI* Rubidium-positron emission tomography myocardial perfusion imaging

### CT-MPI image quality and artifacts

The image quality Likert scores for rest studies was 4 or 5 in 41 (94%), compared to 25 (66%) of stress studies (*p* < 0.05). The reader’s confidence on assessing stress/rest CT-MPI studies was scored as 4 or 5 in 34 (77%) of studies. Likert scores for image quality and reader´s confidence are provided in detail in “Appendix 1”.

There were visible artifacts in 37 (84%) of rest/stress CT-MPI studies. The distribution of artifacts was assessed by segment (17 segments in 44 studies corresponding to a total of 748 segments). In rest studies, there were 105 (14%) segments with artifacts compared to 203 (27%) in stress studies (*p* < 0.001). Beam hardening artifacts were present in 60 (8%) and 43 (6%), and step artifact were present in 30 (4%) and 41 (5%) segments, respectively, on rest and stress studies. The presence of beam hardening artifacts was higher in the right coronary artery 55 (16%) compared to left anterior descending 39 (4%) and circumflex territory 9 (3%) (*p* < 0.0001). The type and distribution of artifacts on rest and stress studies are described in detail in “Appendices 2 and 3”.

### CT-MPI diagnostic performance compared to ^82^Rb PET MPI

**Fig. 3:**
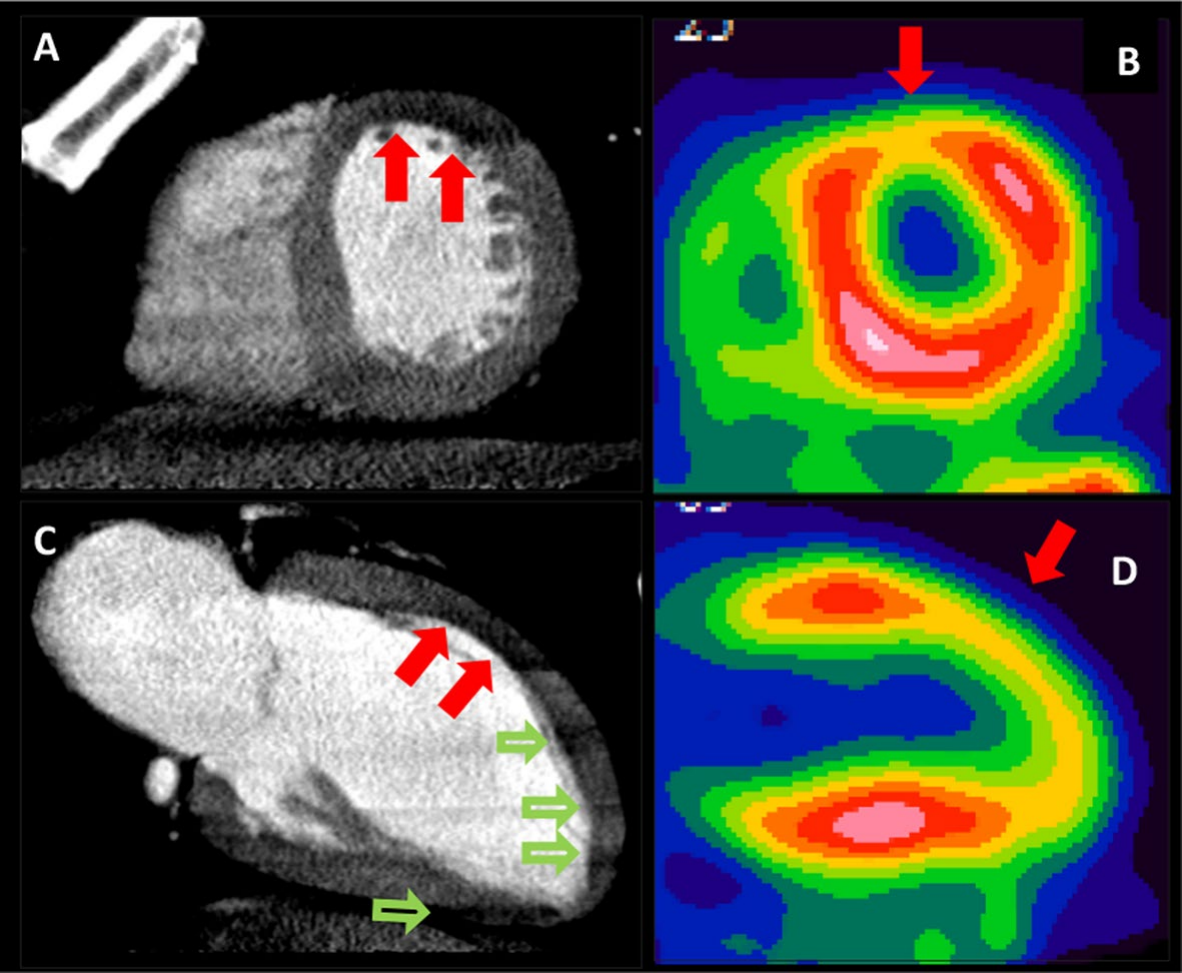
Stress CT-MI and ^82^Rb-PET agreement by visual analysis. Post dipyridamole stress short-axis images of CT-MPI in (**A**, **C**) and ^82^Rb-PET (**B**, **D**). Perfusion defects on post dipyridamole stress CT-MPI in mid anterior segments and corresponding ^82^Rb-PET images in a single patient (red arrows). Note beam hardening artifacts in apical segments in CT images (open green arrows)

There were 26 (59%) patients with reversible perfusion defects on CT-MPI and 18 (41%) patients on ^82^Rb PET (*p* = 0.135) (Fig. 3). There was a moderate agreement between CT-MPI readers in identification of abnormal CT myocardial perfusion on a per-patient basis, with inter-rater agreement weighted kappa of 0.49 (95% CI of 0.28 to 0.70). The sensitivity (SS), specificity (SP), accuracy (AC), for reader A were, respectively, 88.9%, 57.7%, 70.5%, and for reader B were 55.6%, 80.8%, 70.5%. For identification of abnormal CT myocardial perfusion on a per-patient basis, the area under the ROC curve (AUC) was 0.73 and 0.68 (*p* = 0.47), respectively, for reader A and B.

Using the reader´s consensus reading, there was a moderate agreement between CT-MPI and ^82^Rb-PET for identification of abnormal myocardial perfusion on a per patient basis (weighted kappa of 0.44, 95% CI of 0.21 to 0.68).

On a per-patient analysis, CT-MPI compared to ^82^Rb-PET as the reference standard had 88.9% SS, 57.7% SP and 70.5% AC, and AUC of 0.73 (95% CI of 0.58–0.85) (*p* < 0.001). On per-territory basis, CT-MPI had over all 73.1% SS, 65.1% SP, 67.7% AC, and AUC of 0.69 (95% CI of 0.61 to 0.77) (*p* < 0.001) (Table 3).

**Table 3:** Diagnostic performance of CT-MPI on a per patient and per-territory analysis using ^82^Rb PET-MPI as the reference standard

	Per-patient	Per-territory			
	LAD	LCx	RCA	All-territories	
Sensitivity	88.9% [65.3–98.6]	91.7%	80%	44.4%	73.1% [52.2–88.4]
Specificity	57.7% [36.9–76.7]	56.3%	74.4%	62.9%	65.1% [53.4–74.6]
Positive likelihood ratio	2.1 [1.3–3.4]	2.10	3.12	1.23	2.15 [1.5–3.1]
Negative likelihood ratio	0.19 [0.1–0.7]	0.15	0.27	0.88	0.41 [0.2–0.8]
Positive predictive value	59.26% [38.8–77.6]	44%	28.57%	23.53%	33.93% [21.8–47.8]
Negative predictive value	88.2% [63.6–98.5]	94.7%	96.77%	81.48%	90.79% [82.6–96.4]
Accuracy	70.5%	65.6%	75.0%	59.1%	67.7%
Area under the ROC curve	0.73 [0.57–0.85]	0.74	0.77	0.54	0.69[0.60–0.77]

*CT-MPI* computed tomography myocardial perfusion imaging; ^*82*^*Rb PET MPI* Rubidium-positron emission tomography myocardial perfusion imaging; *LAD* left descending coronary artery; *LCx* left circumflex coronary artery; *RCA* right coronary artery; *ROC* receive operating characteristic curve

### CT-MPI and ^82^Rb PET-MPI agreements: measurements of TPD and ischemic TPD

While the SSS, SDS and iTPD were significantly higher (*p* < 0.05) on CT-MPI studies compared to ^82^Rb PET, the SRS and rTPD scores were not different between the two modalities (*p* = 0.105) (Table 4). There was a significant correlation between SDS and iTPD assessed by CT-MPI and ^82^Rb PET (Pearson´s correlation *r* = 0.407, *p* = 0.007, 95% CI 0.122 to 0.631 for SDS and *r* = 0.406, *p* = 0.007, 95% CI 0.120 to 0.6294 for iTPD).

**Table 4:** Comparison of CT-MPI and ^82^Rb PET MPI

	CT-MPI	^82^Rb-PET MPI	*p*
Patients with stress reversible perfusion defects (SDS > 0)	26	18	0.135
Summed Stress Score (SSS)	7.4 ± 7.1	2.5 ± 3.5	< 0.0001
Summed Rest Score (SRS)	1.6 ± 2.8	1.0 ± 2.3	0.105
Summed Difference Score (SDS)	5.8 ± 6.9	1.5 ± 2.3	0.0001
Total Perfusion Deficit Stress (TPDs)	10.9 ± 10.5	3.7 ± 5.2	< 0.0001
Total Perfusion Deficit Rest (TPDr)	2.4 ± 4.0	1.4 ± 3.4	0.105
Ischemic perfusion deficit (iTPD)	8.5 ± 10.1	2.3 ± 3.4	0.0001

SSS, SRS, SDS, TPDs, TPDr, iTPD are expressed as average % ± standard deviation

*CT-MPI* computed tomography myocardial perfusion imaging; *Rb-PET* Rubidium-positron emission tomography myocardial perfusion imaging

**Fig. 4:**
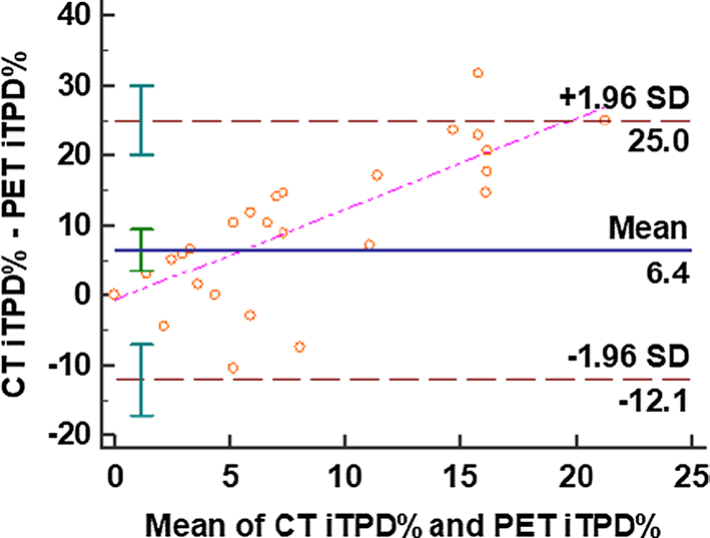
Bland–Altman analysis using visually derived iTPD % demonstrated a mean bias of 6.4; standard deviation of differences: 9.33 and 95% limits of agreement between the two methods: − 12.08 and 24.98. Thus, CT-MPI derived iTPD % overestimated the PET iTPD % by 6.4% on average with the 95% limits as above. The dashed pink line represents the regression line of the differences between CT-MPI and PET iTPD% which shows a positive trend representing that the CT-MPI derived iTPD% overestimation increased with an increasing average of CT-MPI and PET iTPD%. *CT* computed tomography; *MPI* myocardial perfusion imaging; *PET* 82-Rubidium positron emission tomography; *iTPD%* ischemic total perfusion deficit % based on visual semi-quantitative methods

**Fig. 5:**
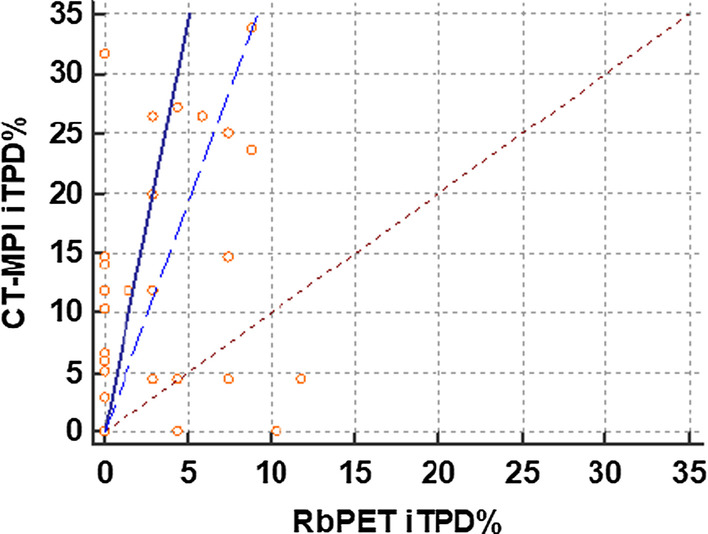
The Passing–Bablok method of comparison demonstrates a proportional bias between the two semi-quantitative visual methods of myocardial perfusion assessment (slope = 6.86, y-intercept = 0, *n* = 43). The blue solid line indicates the slope of CT-MPI iTPD% measurements plotted against the perfect-fit line (dotted red line, slope = 1). The dotted blue line shows the lower limit of the 95% CI which does not include the perfect-fit line and hence, the proportional bias. The proportional bias indicates that the CT-MPI derived iTPD% overestimation of ^82^Rb PET derived iTPD% progressively increases with an increase in perfusion abnormalities as identified by ^82^Rb PET. The CUSUM (cumulative summation) test shows that the observed data does not significantly deviate from a linear model (*P* = 0.53). *CT-MPI* computed tomography myocardial perfusion imaging; ^*82*^*Rb PET* 82-Rubidium positron emission tomography; *iTPD%* ischemic total perfusion deficit % based on visual semi-quantitative methods

Bland–Altman analysis using iTPD scores of CT-MPI and ^82^Rb PET, demonstrated a bias of 6.4%, a standard deviation in differences of 9.3% and, 95% limits of agreement between the two methods of − 12 and 25% (Fig. 4). The regression line of the differences in the two methods showed a positive trend when plotted against the mean of the two measurements. The CUSUM test confirmed linearity (*P* = 0.53) between the two modalities of iTPD measurement, and the Passing–Bablok regression analysis showed good agreement with a proportional bias (slope = 6.86) between iTPD visually derived from CT-MPI and ^82^Rb PET (Fig. 5).

## Discussion

To our knowledge, this is the first study that directly compares myocardial perfusion in static CT-MPI and ^82^Rb PET-MPI using prospective ECG-triggered axial cardiac CT protocol with semi-quantitative visual assessment with a widely available 64-detector CT scanner. This study also provides in-depth information about the CT-MPI image quality and artifacts including their distribution. Our study shows a moderate agreement between static CT-MPI and ^82^Rb PET-MPI (weighted kappa of 0.49, 95% CI of 0.28 to 0.70) and demonstrates good diagnostic performance of CT-MPI on qualitative and quantitative analyses at a per-person (89% SS, 58% SP, 71% accuracy AC) and a per-territory level (73% SS, 65% SP, 67% AC).

Other prior studies have compared CT-MPI and PET-MPI albeit with differing CT and PET methodologies. Williams et al*.* (2017) compared static whole-heart coverage CT-MPI determined perfusion defects with oxygen-15 labelled water PET determined myocardial blood flow (MBF). The authors reported excellent correlation between CT attenuation density and PET determined MBF (*r* = 0.579, *P* < 0.001). Others have utilized either dynamic (Kikuchi et al. 2014) or multiphase (Dantas et al. 2018) CT-MPI protocols to calculate MBF and have shown good correlation with ^82^Rb PET determined MBF. Our study extends the available evidence by demonstrating a moderate agreement between static CT-MPI and ^82^Rb PET-MPI based on a semi-quantitative visual assessment of myocardial segments for reversible perfusion defects.

Our findings lend support to the use of static CT-MPI with a semi-quantitative visual assessment in identifying hemodynamically significant CAD. This is important as a static CT-MPI protocol with a semi-quantitative visual assessment can be easily integrated into a reporting practice and also doesn’t have limitations of a dynamic CT-MPI protocols such as increased radiation exposure (Danad et al. 2016), longer duration of breath hold, need for whole-heart coverage CT scanner, and the need for analytical software for absolute MBF quantification (Koo et al. 2016).

Our study demonstrated a per-patient SS of 88.9%, a SP of 57.7% and 70.5% AC for CT-MPI based on a semi-quantitative visual assessment when compared to ^82^Rb PET-MPI as the reference standard. The per-patient AUC of 0.73 demonstrates a good ability of CT-MPI to discriminate between those with and without myocardial perfusion defects. We found that CT-MPI overestimated perfusion defects in the myocardium as the visually derived SSS and the resultant iTPD from CT-MPI were significantly higher than those derived from ^82^Rb PET-MPI (*p* < 0.0001). While the SRS was not significantly different, the same trend of higher measures was also noted using CT-MPI. Similarly, the Bland–Altman analysis also showed that CT-MPI overestimated perfusion defects and the Passing–Bablok test revealed that this overestimation progressively increased with an increase in perfusion abnormalities as identified by ^82^Rb PET. The overestimation of perfusion defects by CT-MPI is unlikely to be secondary to false positive defects related to artifacts (Blankstein et al. 2009). Of note, artifacts were identified in 27% of patients in our study who underwent stress CT-MPI. Most of these were noise artifacts (11%), and only 6% of beam hardening artifacts, which are known to mimic abnormal myocardial perfusion. The Likert scores for image quality were lower in the stress phase (score 4 or 5 in 66% of stress phase vs 94% of rest phase images) as expected. It seems unlikely that the relatively low number of beam hardening artifacts and image quality limitations could explain the differences in the measures of perfusion defects, as this trend was also noted in the rest studies. It is possible that the CT-MPI stress phase identified an excess of myocardial perfusion defects compared to ^82^Rb PET-MPI because of inherent higher spatial resolution (approximately 0.4 mm of CT compared to 0.7–0.9 mm of PET), higher contrast resolution and dynamic range of CT compared to a nuclear based method. Similar findings were described by Meinel et al*.* (2013), in a study of 55 patients comparing first-pass (static) dual energy CT with SPECT as the reference standard. In this study, almost one-half of defects that were reversible at SPECT were classified as fixed, suggesting that the higher sensitivity of rest CT perfusion could be due to its ability to detect small subendocardial perfusion abnormalities. In fact, it is well described that the presence of perfusion abnormalities at rest CT-MPI that correlate with decreased myocardial flow and myocardial ischemia are probably secondary to the vasodilatory effects of nitroglycerin and iodine contrast inducing a mild hyperemic state (Williams et al. 2017; Meinel et al. 2013; Iwasaki and Matsumoto 2011; Kachenoura et al. 2009; Gupta et al. 2013; Osawa et al. 2014, 2016; Han et al. 2018). However, given a lack of invasive cardiac catheterization correlation in all cases, it is possible that some of these cases could be false positives. Therefore, it may be prudent in clinical practice to correlate CT-MPI findings with CCTA or FFR_CT_ to offset the risk of false positives.

On a per-territory level, the current study demonstrated a good SS of 73.1%, 65.1% SP and 67.7% AC. Of note, this is a small increase in SP from the per-patient value (57.7%) and decrease compared to per-patient SS (88.9%). This is an important result which demonstrates a difference in the diagnostic performance of a semi-quantitative visual assessment of CT-MPI using a per-person or a per-territory methodology. Given the good per-patient SS (88.9%) and the moderately low negative likelihood ratio (0.19) relative to ^82^Rb PET, using CT-MPI with a semi-quantitative visual per-person assessment may be useful in ruling out functionally significant CAD, which would be especially valuable in those with a high pretest probability. On the other hand, a per-territory assessment may be useful in ruling in functionally significant CAD given the moderate SP (65.1%) and positive likelihood ratio (2.15), which would be valuable for those with low pretest probabilities.

It is interesting to note a moderate agreement (weighted kappa 0.49) and a markedly higher SS were recorded for CT-MPI reader A compared to reader B (88.9% vs 55.6%) on a per-patient basis for identification of myocardial perfusion defects. This highlights the issue of inter-reader variability in visual assessment of static CT-MPI. While this result could possibly be explained by the difference in experience of the two CT-MPI readers (Lubbers et al. 2011), it also highlights the need to further investigate visual assessment methods that would reduce inter-reader variability. Nonetheless, the inter-reader variability in our study is reflective of the “real-world” scenario with CT-MPI image readers of varying degrees of experience.

Static CT-MPI addresses a number of the limitations of other MPI modalities. The wide availability of CT scanners compared to PET and CMR, the higher spatial resolution compared to SPECT, and the ability of CT-MPI to simultaneously provide information regarding the functional significance and anatomical assessment of CAD in a short examination duration place CT-MPI favorably compared to the other modalities (Yang et al. 2017). Considering the relative ease with which a visual based semi-quantitative MPI assessment can be integrated into a routine clinical practice, the use of such methodology might prove beneficial to assess the functional significance of CAD. There is emerging data on the added value of integrating CT-MPI with CCTA and FFR_CT_ to provide incremental diagnostic accuracy, specifically in borderline lesions with FFR_CT_ between 0.7 and 0.8 (Coenen et al. 2017; Pontone et al. 2019b). As per the 2020 Society of Cardiovascular Computed Tomography expert consensus, the use of CT-MPI as an adjunct for patients having CCTA is recommended for those at high risk of obstructive CAD or if stenoses have indeterminate functional significance (Patel et al. 2020). Furthermore, stress CT-MPI may be combined with FFR_CT_, as theoretically the former may be more representative of the contribution of epicardial stenosis, microvascular disease and myocardial mass to ischemia.

Multiple studies have compared FFR_CT_ with static CT-MPI in their ability to provide incremental diagnostic value to CCTA in detecting hemodynamically significant stenoses. These studies have shown conflicting results, possibly due to differing CT-MPI acquisition modalities, the type of scanners used and other study limitations including small sample sizes (Pontone et al. 2019a; Yang et al. 2017; Guo et al. 2021; Ihdayhid et al. 2018; Ko et al. 2019). Furthermore, a recent meta-analysis by Celeng et al. (2019), showed similar diagnostic performance of CT-MPI (SS 0.94, 95% CI 0.91–0.97; SP 0.48, 95% CI 0.37–0.59, *n* = 3101) and FFR_CT_ (SS 0.83, 95% CI 0.71–0.92; SP 0.79, 95% CI 0.68–0.87, *n* = 697) on a per-patient basis. CT-MPI can overcome FFR_CT_ limitations such as requirement for off-site interpretation, evaluation of coronary stents and epicardial vessels with complex plaque and anatomy. Furthermore, FFR_CT_ uses CCTA images as boundary conditions for computation fluid dynamic analysis of the coronary tree, and therefore the technique is impaired with the presence of artifacts in the coronary artery images that limit coronary segmentation, such as motion, steps, severe calcification or bypass grafts, factors that do not affect the performance of CT-MPI. Thus, CCTA and CT-MPI can be performed using a 64-detector width MDCT with static low dose protocol, FFR_CT_ evaluation for CCTA datasets may not be feasible in a substantial number of cases (Rochitte and Magalhães 2019).

This study was a single-center, prospective study with a limited sample size and hence, studies in larger cohorts are needed to validate our findings. The large patient size in the study (average BMI 30.8 ± 6.7), the use of 64-detector row CT scanner and a low radiation dose protocol using prospective ECG-gated rest and stress acquisitions may have affected CT results unfavorably. However, despite these technical limitations, our study shows that CT-MPI is feasible and has good accuracy. We used a “rest-first” protocol for CT-MPI which could preclude our ability to identify low-attenuation perfusion defects, since iodine contrast can be retained in small subendocardial infarcts (increasing myocardial density) and theoretically decrease the sensitivity to detect perfusion defects in the stress phase (Koo et al. 2016). However, contrast contamination is likely to affect results for short inter-scan intervals of less than approximately 20 min. In our study an interval of 20 min was used, which should have been sufficient to allow for adequate washout of most of the myocardial contrast. Furthermore, theophyllines were withheld for 24 h more for ^82^Rb PET-MPI than CT-MPI in our study. The difference in duration of withholding theophyllines prior to 82Rb PET-MPI and CT-MPI in our study was driven by our local protocol. As the duration in both cases was 24 h or more (i.e. minimum required), additional 24 h of theophylline free period prior to 82Rb PET-MPI would have not caused any significant effect on study results (Salcedo and Kern 2009). Metoprolol was given orally before the rest CT-MPI. This may have caused some reduction in CT-MPI sensitivity.

Lastly, our study used ^82^Rb tracer for PET, which has a lower image resolution, a lower myocardial extraction fraction compared to Oxygen-15 water tracer (Maddahi and Packard 2014). However, given the need for an onsite-cyclotron for producing Oxygen-15 water tracers, most PET-MPIs are performed using Rb tracers (Takx et al. 2015) thus, reiterating the value of our study for current clinical practice. To our knowledge, nitrogen-13-ammonia PET has not been compared to CT-MPI.

## Conclusions

In conclusion, CT-MPI has high sensitivity and good overall accuracy for the diagnosis of functionally significant CAD using ^82^Rb PET-MPI as the reference standard. CT-MPI may play an important role in assessing the functional significance of CAD especially in combination with CCTA because of its high availability.

## Data Availability

The datasets used and/or analyzed during the current study are available from the corresponding author on reasonable request.

## Appendix 2: CT-MPI artifacts and distribution according to segments and coronary territories

**Table:**

	Rest	Stress	*p**	LAD	LCx	RCA	*P***
No artifacts	643 (86)	545 (73)	< 000.1	717 (81)	222(84)	249(71)	0.002
Artifacts	105 (14)	203 (27)	< 000.1	163 (19)	42(16)	103(29)	0.002
Beam hardening	60 (8)	43 (6)	0.08	39(4)	9(3)	55(16)	< 000.1
Motion	3(0)	40(5)	< 000.1	24(3)	7(3)	12(3)	0.56
Step artifact	30(4)	41(5)	0.18	47(5)	10(4)	14(4)	0.25
Noise	12(2)	79(11)	< 000.1	53(6)	16(16)	22(6)	0.89

*N* = 17 segments × 44 studies = 748; number of findings (%)

*LAD* left descending coronary artery; *LCx* circumflex coronary artery; *RCA* right coronary artery

LAD (1,2,7,8,12,13–17 segments), LCx (5,6,11 segments), and RCA (3,4,9,10 segments) according to 17-segment model

*p** Chi-squared test between Rest and Stress; *P*** Chi-squared test for trend between LAD, LCx and RCA

## Abbreviations

AUC: Area under the receiver operating characteristic curve
CAD: Coronary artery disease
CT: Computed tomography
CCTA: Coronary computed tomography angiography
ECG: Electrocardiogram
FFR: Fractional flow reserve
FFRCT: Fraction flow reserve derived from computed tomography
HU: Hounsfield unit
LV: Left ventricle
MBF: Myocardial blood flow
MPI: Myocardial perfusion imaging
PET: Positron emission computed tomography
ROC: Receiver operating characteristic curve
SPECT: Single photon emission tomography
SRS: Summed rest score
SSS: Summed stress score
TPD: Total perfusion deficit
ICA: Invasive coronary angiography
